# Nonengraftment Haploidentical Cellular Therapy for Hematologic Malignancies

**DOI:** 10.1155/2012/784213

**Published:** 2012-01-18

**Authors:** John L. Reagan, Loren D. Fast, Eric S. Winer, Howard Safran, James N. Butera, Peter J. Quesenberry

**Affiliations:** Division of Hematology and Oncology, Rhode Island Hospital, The Warren Alpert Medical School, Providence, RI 02903, USA

## Abstract

Much of the therapeutic benefit of allogeneic transplant is by a graft versus tumor effect. Further data shows that transplant engraftment is not dependant on myeloablation, instead relying on quantitative competition between donor and host cells. In the clinical setting, engraftment by competition alone is not feasible due to the need for large numbers of infused cells. Instead, low-level host irradiation has proven to be an effective engraftment strategy that is stem cell toxic but not myeloablative. The above observations served as the foundation for clinical trials utilizing allogeneic matched and haploidentical peripheral blood stem cell infusions with minimal conditioning in patients with refractory malignancies. Although engraftment was transient or not apparent, there were compelling responses in a heavily pretreated patient population that appear to result from the breaking of tumor immune tolerance by the host through the actions of IFN**γ**, invariant NK T cells, CD8 T cells, NK cells, or antigen presenting cells.

## 1. Introduction

Allogeneic marrow transplantation exerts much of its therapeutic effect through graft killing of tumor cells. This was established in studies of the effect of donor lymphocyte infusions on relapsed chronic myelocytic leukemia (CML) in marrow transplant patients [1].

Initial insights on the presence of graft versus leukemia came from the work of Thomas and colleagues [2, 3], showing that leukemic relapses were lower in allogeneic transplant patients who had acute and chronic graft-versus-host disease (GVHD) as compared to those who did not develop these complications. Furthermore, patients with both acute and chronic GVHD had lower relapse rates than those with either acute or chronic GVHD alone [4, 5]. There was an increased leukemic relapse rate in identical twin marrow transplants as compared to allogeneic transplants [2, 6]. T-cell depletion from allogeneic marrow infusions decreased acute and chronic GVHD, but increased the rate of relapses. In a similar fashion, cyclosporine immunosuppression after allogeneic transplantation increased leukemic relapses, and discontinuation of the drug at the first sign of a relapse could induce a remission [7–9].

The most direct evidence of a cellular immune attack against leukemic cells was provided by Kolb and colleagues [1]. They demonstrated high rates of durable remissions in relapse CML with the simple infusion of donor lymphocytes. Donor lymphocyte responses after relapse from allogeneic transplant were also seen in patients with AML, ALL, MDS, polycythemia vera, lymphoma and myeloma [10, 11]. Thus cellular therapy in patients with a variety of marrow malignancies has been established and appears to be mediated by T lymphocytes although other cell types might also be involved.

## 2. Origins of Nonmyeloablative Transplantation

These studies suggested that cellular approaches without toxic myeloablative therapy might be effective treatment for many marrow malignancies. General dogma had been that myeloablative treatment was needed to open space so that marrow cells could engraft. However, Micklem et al., in 1968, demonstrated that engraftment into nonirradiated hosts was feasible, obtaining up to 8.5% T6T6 donor cells in CBA host marrow at 3 months after transplantation of 20 million marrow cells from normal T6T6 donors [12]. This work was extended by others [13–15].

The studies by Brecher and colleagues formed the basis for extensive studies in our laboratory on engraftment into nonmyeloablated mice, which in turn led to applications of nonmyeloablated transplantation in humans. Studying male BALB/c marrow engrafted into female BALB/c mice with no cytoreductive therapy, we determined that engraftment was essentially quantitative resulting from competition between infused cells and host cells. Evaluated mice which had received 5 consecutive intravenous injections of 40 million marrow cells showed continued engraftment in marrow at 21–25 months after transplant ranging from 15% to 42%. Patterns of engraftment were similar for thymus and spleen [20]. Further studies showed that high numbers of cells given in one injection gave equal values to engraftment to the same number of cells divided over multiple injections [21–23]. Critical features of marrow engraftment were approached by determining total murine marrow cellularity and the observed engraftment percentages when 40 million marrow cells were infused (72 mice) and comparing those with theoretical outcomes [24]. Total cellularity in BALB/c mice was 530 ± 20 million cells, stable from 8 weeks to 1 year of age. Using this data, a theoretical model of infused marrow (40 million cells) replacing or adding to host marrow gave values of 7.5 and 7.0%, respectively; the observed 8-week engraftment of 40 million male BALB/c marrow cells into female hosts (72 mice) was 6.91 ± 0.4%. This indicated that syngeneic engraftment was determined solely by stem cell competition, not by “opening space”. A summary of the above is depicted in List 1.

**List 1: Engraftment Into Normal Untreated Mice**

- High levels of chimerism seen in marrow, spleen, and thymus.
- Chimerism persistent out to 2 years.
- Chimerism was multilineage.
- All donor cells appeared to have been engrafted. Final readouts were determined by competition between host and donor cells.

These data also indicated that so-called niches are not limiting as to engraftment, although there are clearly areas favoring specific differentiation pathways. Most “niche” studies need to be reconsidered since they have been carried using a dormant purified murine marrow stem cells, which do not appear to be representative of the true marrow long-term repopulating stem cell.

## 3. The Role of Reduced Intensity Conditioning

These data formed a base for engraftment into non-myeloablated mice. However, engraftment into non-myeloablated mice required large number of marrow cells, which would be difficult to obtain in a clinical setting. Accordingly, we investigated whether minimal myeloablation with low doses of irradiation would be an effective engraftment strategy [25]. In these studies, we demonstrated that exposure of BALB/c mice to doses of irradiation that cause minimal myeloablation (50–100 cGy) gave high levels of donor chimerism, such that relatively small numbers of marrow cells (10–40 million) can give donor chimerism in the 40–100% range. These doses of irradiation turned out to be minimally myeloablative but quite stem cell toxic. Engraftable stem cells measured at 8 weeks after engraftment from mice exposed to 100 cGy whole body irradiation were reduced to 8.6 ± 3% of marrow from nonirradiated mice. At 6 months, the reduction was still present, 21 ± 7% [26]. These data provided us with a stem cell toxic nonmyeloablative approach for therapeutic transplantation. Engraftment with reduced intensity conditioning is summarized in List 2.

**List 2: Minimal Myeloablation and Engraftment**

- 50–100 cGy gives relatively high levels of engraftment with 10–40 million murine marrow cells.
- These levels of irradiation are minimally myelotoxic with mild effects on blood counts.
- These levels of irradiation are very stem cell toxic.
- A stem cell toxic nonmyeloablative approach poses interesting therapeutic possibilities.

## 4. Presence of Engraftment

However, it was not clear if this would hold for allogeneic engraftment. Accordingly, we investigated engraftment into non or minimally myeloablated allogeneic mice. We felt that low-dose irradiation might avoid the cytokine storm which appeared to be involved in GVHD, and that relatively high levels of marrow cells might overcome rejection. However, we found that we could not obtain engraftment using 100–300 cGy and 40 million cells in H-2 mismatched B6.SJL to BALB/c marrow transplants. Clearly, immune barriers existed.

We then tried antigen preexposure and costimulation blockade in this setting. Ten million B6.SJL spleen cells were infused into BALB/c mice 10 days prior to transplantation, and anti-CD40 ligand antibody was given immediately prior to the spleen cell infusion and thereafter on days −7, −3, 0, and +3. On day 0, 40 million B6.SJL whole marrow cells were infused into the BALB/c hosts which had received 100 cGy within 4 hours of marrow infusion. Stable multi-lineage chimerism at levels between 30–40% was achieved in the great majority of mice at 1.6 mg anti-CD40 ligand monoclonal antibody per injection out to 64 weeks after transplantation [27]. There was no GVHD, and mice were tolerant to donor B6.SJL skin grafts.

We subsequently evaluated whether we could obtain significant marrow engraftment in this H-2 mismatched model by first establishing microchimerism to set the stage for macrochimerism. We showed that establishment of microchimerism (0.5–3.8%) with subsequent infusions of 40 million marrow cells on weeks 12, 14, and 16 or weeks 3, 4, 5, and 6 with injections of anti-CD40 ligand antibody, but without irradiation or spleen cell injection, resulted in significant engraftment [28]. In the latter schedule, engraftment was 17.9 ± 1.2% at 24 weeks. Thus, we could obtain significant engraftment with scheduled marrow cells and costimulator blockade. Giving the same number of total cells on day 0 did not result in significant chimerism. Therefore, a scheduling effect was critical for these results in mismatched allogeneic murine transplants. These results are different from the results in syngeneic transplants where scheduling appears to have no effect, rather total cell dose determines engraftment levels. These studies are summarized in List 3.

**List 3: Minimal to No Myeloablation with Mismatched Allogeneic Marrow Transplantation**

- 100 cGy, spleen cell exposure and CD40 ligand antibody give significant chimerism with 40 million cells given in one injection.
- A scheduled engraftment with 40 million cells given multiple times gives significant engraftment with only costimulator blockade.

## 5. Clinical Trials

These studies plus the evolving studies on the impact of donor lymphocyte infusions in clinical transplant set the stage for human trials using allogeneic or haploidentical peripheral blood infusions in patients with refractory cancers. Patients with refractory cancers were treated with 100 cGy total body irradiation followed by infusion of nonmobilized apheresed allogeneic peripheral blood cells. Twenty-five patients were enrolled [29]. Transplants were with either HLA matched or 1/6 mismatched, one antigen mismatched family donors, or 4–6/6 antigen matched umbilical cord blood donor cells. Seven patients with solid tumors received a sibling transplant and 6 received a cord blood transplant; none achieved donor chimerism but 1 treated at the higher-dose level of 1 × 10^8^ CD3+ cells/kg had a transient nodal response.

Twelve patients with hematologic malignancies were treated; eleven receiving sibling donor cells. Nine of these eleven achieved donor chimerism ranging from 5–100%. In this group, there were four complete remissions and of these, three had 100% chimerism. Two developed GVHD with one dying of liver GVHD and the other succumbing to disease relapse. One patient with AML and 100% chimerism achieved a complete remission that required reinfusion of donor cells due to persistent pancytopenia. Interestingly, another patient with a large cell lymphoma and only transient 5% chimerism for one week developed a sustained complete remission for at least 42 months after transplantation. This particular patient was heavily pretreated with salvage chemotherapy and radiotherapy for relapsed disease following autologous transplantation. A similar remarkable response was seen without engraftment in a patient with CLL who witnessed a 75% decrease in lymphadenopathy, despite no evidence of chimerism.

In order to expand donor base, we next evaluated the safety and efficacy of haploidentical transplantation in a phase I/II nonimmunosuppressive nonmyeloablative setting [16]. A total of 41 patients with relapsed refractory cancers received 100 cGy total body irradiation along with an infusion of 1 × 10^6^ to 2 × 10^8^ CD3+ cells per kilogram. Twenty nine patients received the highest dose of 2 × 10^8^ cells/Kg. A post-infusional syndrome termed “haploimmunostorm” was seen at the two highest cell levels. This consisted of fever with a median onset of 14 hours after cell infusion and malaise, liver function test alterations, morbilliform rash, and diarrhea to varying degrees. Skin biopsies were negative for GVHD. This syndrome rapidly responded to steroid administration and was probably a variant of cytokine storm.

In all, there were 26 patients with hematologic malignancies with 14 responses, 7 of which were major. Two of six patients with lymphoma remained free of disease at 76 and 82 months, respectively. There were 5 durable complete responses and 4 partial responses in 13 patients with AML. All responses occurred outside the donor chimerism with sensitivity of 1–5% in the chimerism determinations. There were no responses with the solid tumor patients, and two patients who converted to total chimerism died, one of GVHD. Altogether, these results indicated that there was significant antitumor activity in the setting of rejection of infused marrow cells, some of which were clinically significant. Presently, the mechanisms underlying this are unclear, but we favor a breaking of host tolerance to tumor cells. These results are summarized in List 4.

**List 4: Allogeneic and Haploidentical Cellular Therapy of Hematologic Malignancies**

- HLA-matched engraftment in nonmyeloablated setting showed tumor responses associated with chimerism.
- Haploidentical infusions in nonmyeloablated setting caused responses in lymphoma and AML patients without chimerism.

Clearly, nonengraftment (at least maxi-engraftment) presents a potentially new direction in therapy of refractory hematologic malignancies. An interesting study from China [17] in elderly patients indicated that adding haploidentical cell infusions to chemotherapy markedly improved results with no GVHD, but with persistent donor microchimerism. Prolonged survival in patients who do not achieve engraftment has been reported elsewhere in patients with acute lymphocytic leukemia, non-Hodgkin lymphoma, Hodgkin lymphoma, myelodysplastic syndrome, and multiple myeloma [18, 19].

These results summarized in Table 1 suggest that studies of hematologic malignancies in which marrow rejection is the goal are indicated to more fully evaluate both clinical efficacy and underlying mechanism.

## 6. Proposed Mechanism of Action

The underlying mechanism of cellular therapy efficacy is only recently coming to light and is thought to include everything from the growth factors employed for cell collection to the actual composition of the cells themselves. Clearly T cells are involved in this process; however, neither the bystander killing effect nor the T cell receptor cross reactivity to allogeneic antigens with tumor antigens is thought to play a role. Central to the development of a graft versus leukemia or host versus leukemia response is the underlying regulation between the development of a T_H_1 response and a graft or host versus tumor effect (and with it the potential for acute or chronic GVHD) and a T_H_2 response that allows for tolerance of the graft and prevents GVHD. Clinical trials suggest that an initial engraftment of donor cells followed by the host rejection of this engraftment is essential to developing a host versus tumor response. Based on this concept, Sykes and colleagues developed a mixed chimeric bone marrow murine model to more fully understand the mechanism of this response. The mixed chimera developed represents the initial infusion and partial engraftment of haploidentical cells. They are then able to model the rejection of these haploidentical cells through the infusion of recipient murine leukocytes (RLI). RLI infusion then induces a host versus graft (HVG) reaction that results in an antitumor response by the host's immune system to malignancies present within the host which they have been able to further characterize. This response requires a complex interaction between interferon-*γ* (IFN-*γ*), CD8 T cells, invariant NK-T cells (iNKT), NK cells, and antigen presenting cells.

Early work by this group first disclosed the importance of IFN-*γ* in the development of an antitumor response. They further determined that IFN-*γ* derived from the recipient was essential for the development of an antitumor response [30]. Overall, a robust cytokine response was seen clinically when mixed chimerism is lost. Although many other cytokines are involved in this response, experiments have shown that IFN-*γ* generation was crucial for an antitumor response. IFN-*γ* is also important in the allogeneic transplantation graft versus leukemia effect [31]. Later experiments determined that IFN-*γ* production initially is through RLI CD8+ T cells by leukocytes present within the RLI fraction as well as non RLI recipient leukocytes. The presence of RLI CD8+ T cells and non RLI recipient CD4+ cells is crucial for the development of antitumor responses to RLI [32]. Also involved in the anti-tumor response are recipient iNK-T cells with the anti-tumor effects of these cells distinct from RLI CD8+ T cells. Following RLI, iNK-T cells become activated and in turn activate both recipient NK cells and dendritic cells [33]. The end theoretical result is the immune activation and subsequent breaking of host tolerance to tumor.

In addition to the cells themselves, the initial collection of cells for infusion and methods therein may play a role. For example, G-CSF has been shown to promote T_H_2 differentiation and T regulatory cell proliferation, while also expanding the pool of immature antigen presenting cells. All of these measures help prevent the development of GVHD, while not inhibiting the cytolytic T cell graft versus tumor effect. A further increase in GVT is seen when G-CSF is pegylated, which leads to activation of invariant NK cells. These cells are thought to play a role in the development of cytotoxic response through cytokine release that leads to further recruitment of NK cells and CD8+ CTLs [34]. Other cell types such as CD4+/CD25+ T regulatory cells (T-regs) have been shown to play a role in the donor and recipient cell interactions in haploidentical transplant where engraftment occurs [35, 36]. Tregs appear to have a role in host tolerance and allow for an increase in engraftment with a decrease in GvHD. The role Tregs have in the nonengraftment setting is still unclear.

## 7. Conclusions

As outlined above, cell infusional treatment of leukemia and lymphoma is not completely dependent on graft versus tumor/leukemia effect. Rather, rejection of the graft itself appears to reeducate the host's immune system to recognize the tumor, thereby creating a host versus tumor effect. This method is not without its side effects in the form of a cytokine storm involving IFN-*γ* but does bypass the morbidity and mortality that develop from graft versus host disease. An additional advantage of non-engraftment haploidentical transplantation over allogeneic transplantation is the availability of donors. Family members are readily identified as potential donors making this a readily available treatment modality. The overall keys to the future of nonengraftment haploidentical transplantation reside in better understanding of the degree of chimerism, if any, necessary for a reaction as well as the timing and role of host effector cell stimulation. The potential result is the ability to harness the host's immune system in order to provide an effective therapeutic modality to eradicate malignancy. Future studies should help decipher the underlying interaction that occurs between host and donor cells. Key questions that remain are the amount and type of conditioning regimen as well as the degree of antigenic mismatch required to stimulate a host versus tumor response in vitro and in vivo. Potential further clinical trials could focus on decreasing or eliminating radiation or chemotherapy conditioning in addition to examining the effects of complete HLA mismatch in order to further remove any chance of engraftment and with it GvHD development. Moreover, future trials may also explore the role of multiple cellular infusions spaced out in a treatment plan to possibly elicit a more robust response.

## Figures and Tables

**Table 1:** Summary of nonengraftment cellular therapy results.

Author	Number of patients	Conditioning regimen	Number of CD3 cells infused	Response rate (CR/PR)
Colvin et al. [16]	19 patients (AML/NHL only)	100 cGy TBI	1 × 10^6^–2 × 10^8^/kg	37% (26%/11%)

Guo et al. [17]	30 patients (AML)	Mitoxantrone 8–10 mg/m^2^ (3 days) and cytarabine 150 mg/m^2^ (7 days)	0.5–2.6 × 10^8^/kg	80% (80%/NR)

*O'Donnell et al. [18]	4 patients (ALL, MDS)	Fludarabine 30 mg/m^2^, 200 cGy TBI, Post transplant ± Pretransplant cyclophosphamide	2.6–3.8 × 10^7^/kg	75% (75%/0%)

*Dey et al. [19]	22 patients (NHL, HL, Multiple Myeloma)	Cyclophosphamide 50 mg/kg (3-4 days), ATG 15–30 mg/kg (3-4 days), and anti-CD 2 monoclonal antibody	1 × 10^7^–2.98 × 10^8^/kg	41% (18%/22%)

*Only patients who did not engraft in this study are included in the results. AML= acute myeloid leukemia, NHL= non-Hodgkins lymphoma, MDS= myelodysplastic syndrome, ALL= acute lymphocytic leukemia, HL= Hodgkins lymphoma.
